# RNA processing in the minimal organism *Nanoarchaeum equitans*

**DOI:** 10.1186/gb-2012-13-7-r63

**Published:** 2012-07-18

**Authors:** Lennart Randau

**Affiliations:** 1 Max-Planck-Institute for Terrestrial Microbiology, Karl-von-Frisch Strasse 10, 35037 Marburg, Germany

## Abstract

**Background:**

The minimal genome of the tiny, hyperthermophilic archaeon *Nanoarchaeum equitans *contains several fragmented genes and revealed unusual RNA processing pathways. These include the maturation of tRNA molecules via the trans-splicing of tRNA halves and genomic rearrangements to compensate for the absence of RNase P.

**Results:**

Here, the RNA processing events in the *N. equitans *cell are analyzed using RNA-Seq deep sequencing methodology. All tRNA half precursor and tRNA termini were determined and support the tRNA *trans*-splicing model. The processing of CRISPR RNAs from two CRISPR clusters was verified. Twenty-seven C/D box small RNAs (sRNAs) and a H/ACA box sRNA were identified. The C/D box sRNAs were found to flank split genes, to form dicistronic tRNA-sRNA precursors and to be encoded within the tRNAMet intron.

**Conclusions:**

The presented data provide an overview of the production and usage of small RNAs in a cell that has to survive with a highly reduced genome. *N. equitans *lost many essential metabolic pathways but maintains highly active CRISPR/Cas and rRNA modification systems that appear to play an important role in genome fragmentation.

## Background

*Nanoarchaeum equitans *is a 400 nm small archaeon isolated from hot submarine vent microbial communities whose growth relies on its attachment to the cell surface of the archaeon *Ignicoccus hospitalis *[1]. Phylogenetic analyses based on its unusual ribosomal RNA sequences placed *N. equitans *into a novel phylum termed 'Nanoarchaeota'. However, different phylogenetic studies focused on ribosomal proteins and concluded that *N. equitans *represents a member of a fast-evolving euryarchaeal lineage related to the Thermococcales [2]. The genome sequence of *N. equitans Kin4-M *revealed a minimal, compact genome of only 490 kilobases and an extremely high gene density with little noncoding DNA or pseudogenes [3]. This highly reduced genome lacks almost all known genes for the synthesis of amino acids, nucleotides, cofactors, and lipids. Conserved operonic structures are absent and an unusually high number of genes is found in split variations [3,4]. Examples of such splits are the two open reading frames encoding domains of the alanyl-tRNA synthetase or the reverse gyrase [3]. Other unusual features concern the processing of RNA molecules. *N. equitans *was the first organism shown to require the assembly of tRNA halves to generate six essential functional tRNA isoacceptors [5]. A heteromeric splicing endonuclease generates these mature tRNAs via an unusual *trans*-splicing reaction [6-8]. *N. equitans *is also the only currently identified organism that can survive without an RNase P molecule [9-11]. RNase P is an otherwise universal ribonucleoprotein complex that mediates the removal of 5' leaders in pre-tRNAs. The absence of both RNA and protein components of RNase P is compensated by genomic rearrangements that resulted in a removal of 5' leader sequences from all *N. equitans *tRNA genes, ensuring proper transcription initiation conditions.

The loss of many essential pathways has to be compensated by the transfer of metabolites between *N. equitans *and *I. hospitalis *[12]. It is assumed that direct cell-cell surface contacts as well as interconnections via thin fibers fulfill this purpose [13]. The *N. equitans *genome encodes a fairly complete set of proteins for replication, transcription and translation. In addition, surprisingly extensive sets of genes with proposed roles in DNA repair and RNA modification are annotated. Finally, two clustered regularly interspaced short palindromic repeats (CRISPR) arrays and a complete set of CRISPR associated (Cas) proteins are present. These systems are mainly characterized as adaptive antiviral defense systems even though the viral threat towards *N. equitans *is not known [14,15]. In this study, RNA-Seq deep sequencing methodology was used to analyze the RNA components involved in the processing and maturation of tRNAs, rRNAs and CRISPR RNAs (crRNAs) to obtain insights into the usage of small RNA molecules in an organism that has to survive with a minimal and condensed genome.

## Results and discussion

### Abundance of RNA species

**Figure 1:**
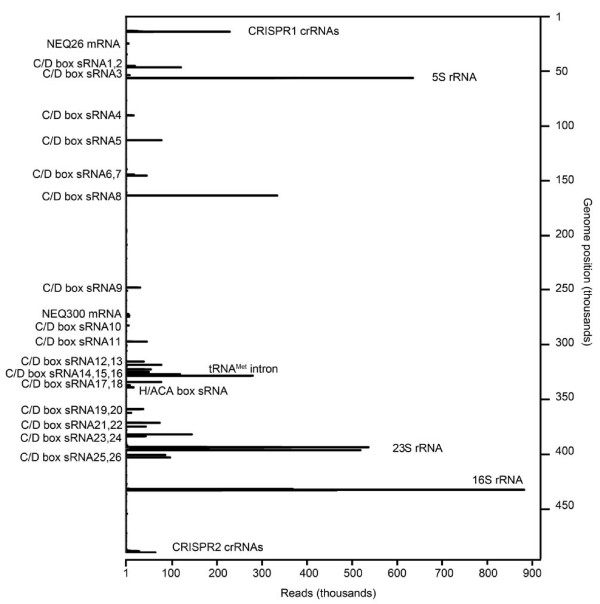
**RNA abundance in *N. equitans***. Illumina HiSeq2000 sequencing reads mapped to the *N. equitans *reference genome (GenBank: NC_005213, 490885 bp) highlight the abundance of crRNAs and C/D box sRNAs.

RNA-Seq methodology was used to obtain a global overview of the production and processing of small RNA molecules in *N. equitans*. Depending on the chosen method of RNA isolation, library preparation and employed RNA sequencing platform, different RNA species are selectively enriched [16]. To obtain the most complete picture of the small RNA diversity present in the *N. equitans *cell, small RNAs were enriched but not depleted of ribosomal RNAs. All RNA samples were treated with T4 polynucleotide kinase and tobacco acid pyrophosphatase before adapter ligation. These steps were employed to ensure that RNAs with 5'-triphosphate or 5'-OH termini and 2',3'-cyclic phosphate termini were captured. Illumina HiSeq2000 sequencing was employed, which yielded over 12 million reads that could be mapped onto the *N. equitans *genome (Additional file 1). Analysis of the abundance of different RNA species verified that all expected different types of RNA species were detected in the RNA-Seq data, including rRNAs, tRNAs, tRNA half molecules, small RNAs (sRNAs) and crRNAs. Nevertheless, only a surprisingly small amount of mature tRNAs was detected and sequencing reads (usually less than 1,000 reads) mapped to fragments of tRNA genes. This observation is thought to exemplify the challenges that highly structured and heavily modified RNA sequences pose for reverse transcriptase enzymes. Most obtained sequencing reads (approximately 8.5 million reads) mapped to the genes encoding the 5S, 16S and 23S rRNAs followed by several highly abundant C/D box sRNAs and crRNAs (Figure 1). The analysis of these two small RNA species is detailed below. Even though the RNA isolation approach was not set up to purify mRNAs, the depths of the obtained sequencing results allowed the mapping of abundant fragmented mRNA reads. Most mRNA fragment reads (approximately 119,000 reads) were mapped to the two genes NEQ300 (S-layer protein) and NEQ026 (protein with unknown function). Both gene products were also identified by whole-cell proteomics to be among the six most abundant *N. equitans *proteins [12]. The only identifiable homolog of NEQ026 is found in *Thermofilum pendens *(arCOG06945) and linked to a putative amino acid permease. This observation provides a starting point for the analysis of the highly abundant protein NEQ026 potentially being involved in the uptake of amino acids from *I. hospitalis*.

### Maturation of tRNA molecules

**Figure 2:**
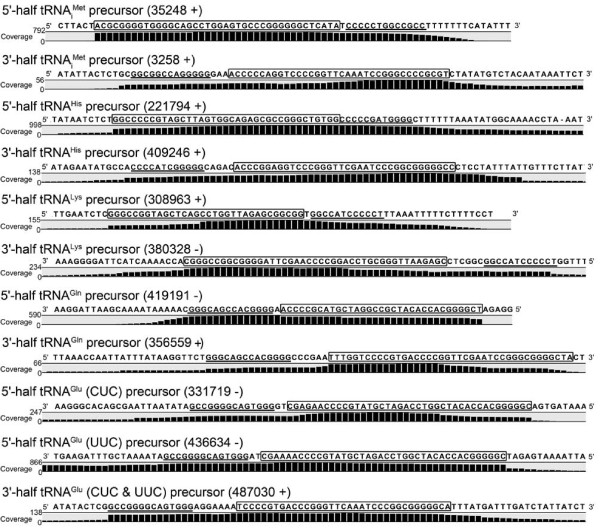
**Identification of tRNA half precursors for tRNA splicing in *trans***. The observed *N. equitans *genome coverage of Illumina HiSeq2000 sequence reads pinpoints the termini of tRNA half precursors. Annotation of the tRNA precursors containing sequences that assemble into the final tRNA (boxed) and GC-rich reverse complementary stretches that identify the matching half (underlined) are taken from [17].

*N. equitans *was the first organism to be identified to generate six tRNA isoacceptors via a *trans*-splicing reaction using tRNA half molecules. Earlier studies characterized the mature spliced tRNA molecules in the cell, yet only a single tRNA half transcript could be identified [5,17]. The depth of the available RNA-Seq data allowed the identification of all 11 tRNA halves in the *N. equitans *cell. The tRNA half molecules contain the sequence that will fold into the mature tRNA body and a GC-rich stretch that is complementary to a sequence only found in the matching tRNA half. These sequences are proposed to facilitate the identification of the matching halves upon which the tRNA body folds [5]. Subsequently, the concerted action of a heterotetrameric splicing endonuclease and an RNA ligase generates *trans*-spliced mature tRNAs. The termini of all tRNA halves were identified (Figure 2). The 5'-termini of the tRNA precursors are more defined than their 3' termini and contain the purine residue required for the proper initiation of transcription. In most cases the tRNA precursor transcripts do not extend beyond the GC-rich stretch. This region is only extended by two nucleotides (GC) for the 3' tRNA^Met ^half, by one nucleotide (A) for the 3' tRNA^His ^half and shortened by one nucleotide (A) for the 3' tRNA^Lys ^half. In some cases, the obtained tRNA precursor sequences merge with the mRNA of the adjacent gene. One example is the 5' tRNA^His ^half that is located directly upstream of the gene encoding the valyl-tRNA synthetase and that defines its 5' untranslated region. It is tempting to speculate that this structured tRNA half might play a role in mRNA stability or regulation of valyl-tRNA synthetase synthesis. The 5' tRNA^Glu ^(UUC) half and 3' tRNA_i_^Met ^half sequences extend into adjacent genes of different orientation, which might cause difficulties for proper termination signals. These sequences indicate that a defined 3' end might not always be a necessity for the assembly of tRNA halves into functional tRNA molecules. Finally, both 5' tRNA halves for the tRNA_i_^Met ^and the tRNA^His ^already contain the additional -1 base that leads to an extended acceptor stem in the mature tRNA.

### Absence of RNase P

Potential low abundance structural RNA molecules were searched in intergenic regions but the otherwise universal RNase P RNA molecule could not be detected, which is in agreement with previous studies [9]. Genomic rearrangements compensate for the loss of RNase P and ensure that all tRNAs start with a purine residue directly at the transcription initiation site. The RNA-Seq data allowed us to analyze the 5' tRNA termini and verified the absence of leader sequences. One interesting example is tRNA^Tyr^, which requires a C1 base for its recognition by the tyrosyl-tRNA synthetase. However, without RNase P, such tRNAs could not start with a pyrimidine residue and it was reported that an unsual G-1 extension solves the need for both a C1 base and a purine residue at the transcription start [9]. As this unique acceptor stem is direct evidence for the absence of RNase P, the RNA-Seq reads were mapped to (i) the tRNA gene containing an intron and (ii) the mature intron-less tRNA. While there were significantly less reads detected for the mature tRNA due to problems of reverse transcription of a fully modified tRNA, the vast majority of all sequences that mapped to the tRNA^Tyr ^locus did contain the G-1 extension.

### Processing of CRISPR RNAs

**Figure 3:**
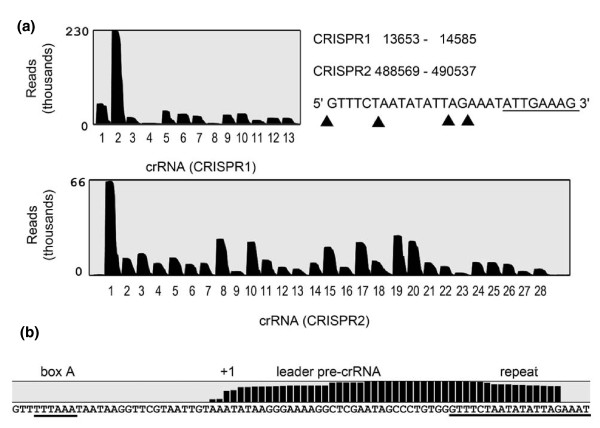
**Identification of CRISPR RNA processing**. **(a) **The crRNA sequencing reads were mapped to the two *N. equitans *CRISPR clusters to determine the abundance of individual crRNAs. Processing occurs within the repeat elements, generating crRNAs with a 5'-terminal ATTGAAAG 8 nucleotide tag (underlined) and gradual trimming of the 3'-terminal tag. Arrows indicate hotspots for trimming. **(b) **Sequencing reads upstream of the first repeat within the identical leader sequence of both CRISPR arrays indicate the transcription start site (+1) and a potential promoter TATA box (box A) element.

*N. equitans *contains two CRISPR clusters whose crRNAs were found to be highly abundant in the cell. It should be noted that both CRISPR clusters are annotated in the reverse orientation in popular CRISPR databases [18,19]. These crRNAs consist of individual spacer sequences that were shown in other organisms to correspond to viral or conjugative plasmid fragments that were incorporated into the CRISPR during an earlier attack by this mobile genetic element and provide resistance against repeated attacks via base complementarity [15]. Viruses that attack *N. equitans *are not known. Nevertheless, the reduced genome still allows the presence of 41 different spacer sequences distributed onto two CRISPR clusters. The CRISPR clusters are transcribed and cleaved within identical 28 bp repeat sequences. Processed crRNAs were detected for all spacers and contained a 5'-terminal 8 nucleotide tag containing the 3'-terminal repeat nucleotides ATTGAAAG that is usually generated by Cas6 cleavage (Figure 3). The 3' ends are gradually shortened and suggest 3' terminal exonucleolytic degradation. The abundance of crRNAs varies drastically, with the highest abundance for spacer 2 of CRISPR cluster 1 and spacer 1 of CRISPR cluster 2. This accumulation of crRNA at the 5' terminus of the CRISPR cluster is in agreement with previous observations that spacers in close proximity to the promoter region within the leader region of the CRISPR cluster represent the most recent attacks [20]. The drastically reduced abundance of some spacers can be an effect of reduced stability of the crRNA or inefficient pre-crRNA processing. In addition, recent work in *Sulfolobus solfataricus *identified a correlation between abundance and RNA folding propensity [21]. The low abundant crRNA 4 of CRISPR cluster 1 contains a spacer that is considerably longer than all other spacers in the cluster, which might pose challenges for the crRNA maturation machinery. Adjacent to both CRISPR clusters, a leader region was identified whose sequence is identical for 130 nucleotides upstream of the first repeat. Thus, both CRISPR clusters possess identical promoters and transcription starts in both cases at an adenosine residue 33 nucleotides upstream of the first repeat. The box A region of archaeal promoters is usually located at a fixed distance from the transcription start site [22] and a consensus 5'-TTTAAA-3' sequence was indeed identified -27 nucleotides upstream of the transcription start. Identical promoter and leader regions explain the comparable rates of crRNA production from both CRISPR clusters and suggest a recent duplication event. The CRISPR/Cas system of *N. equitans *belongs to the recently defined type I systems with Cas3 (NEQ022) as the protein that catalyzes the degradation of the viral DNA [23,24]. All universal proteins Cas1 (NEQ017), Cas2 (NEQ016) and Cas4 (NEQ021) proposed to be involved in the integration of spacers are present. Finally, NEQ018, Cas7 (NEQ019) and Cas5 (NEQ020) are proposed to form the Cascade complex that delivers the crRNAs to the DNA target. A Cas6 enzyme cannot easily be identified, which might indicate its sequence divergence from known Cas6 enzymes.

### Identification of C/D box and H/ACA box sRNAs

**Figure 4:**
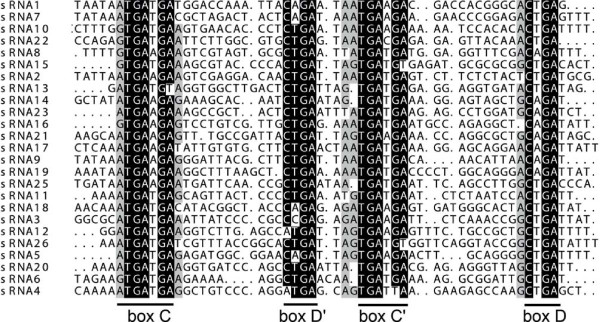
**Identification and alignment of C/D box sRNAs**. Twenty-six C/D box sRNAs, each covered by over 3,000 mapped sequencing reads (Additional file 2) were identified. All sRNAs contain conserved box C/D and box C'/D' elements. The permuted sRNA24 is detailed in Figure 7.

**Figure 5:**
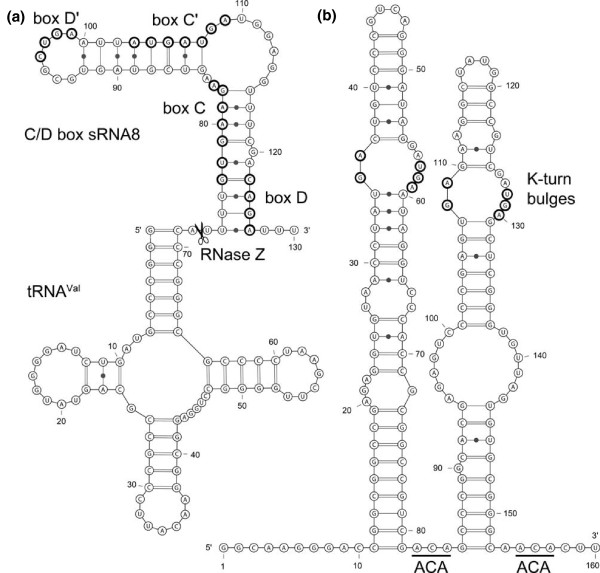
**Structure prediction for a dicistronic tRNA-C/D box sRNA and a H/ACA box sRNA**. **(a) **The most abundant C/D box sRNA, sRNA8, is located immediately downstream of tRNA^Val^(TAC). The tRNA secondary structure was obtained with tRNA-Scan-SE [41] and a possible C/D box sRNA8 secondary structure was computed with mfold [40]. The box C/D and box C'/D' elements and the proposed RNase Z cleavage site are indicated. **(b) **Possible secondary structure (mfold [40]) for the single identified H/ACA box sRNA. Two ACA motifs and two identical k-turn bulges with potential GA-GA pairs are indicated.

*N. equitans *maintains a large arsenal of RNA modification enzymes, and during genome annotation, 14 small nucleolar (sno)-like RNAs that serve as guide RNAs for the methylation of rRNAs were described [3]. The data showed that these snoRNAs (C/D box sRNAs in archaea) are highly abundant in the cell (Figure 1). Furthermore, the sequences for 26 C/D box sRNAs could be identified (Figure 4). All 14 C/D box sRNAs predicted by the snoscan algorithm [25] were verified and five predictions classified as 'questionable' partly overlapped with the identified transcripts. Seven additional C/D box sRNAs were identified for which computational predictions failed. The alignment of all identified C/D box sRNAs highlights their compact and conserved arrangement as well as the characteristic box C/D and box C'/D' sequence motifs (Figure 5). The 5' and 3' ends of these RNAs are not base-paired. Putative 2'O-ribose-methylation targets were predicted by the algorithm PLEXY [26] (Additional file 2). The proteins fibrillarin (NEQ125) and NOP56 (NEQ342), which associate with these C/D box sRNAs and guide methylation, are present in the cell. The set of archaeal methyltransferases include an enzyme that is only found in Thermococcales and *N. equitans *and uses S-adenosylmethionine as a cofactor to catalyze the formation of 5-methyl uridine in tRNAs and rRNAs [27]. A newly identified C/D box sRNA (sRNA2) overlaps with the gene for this methyltransferase (NEQ053), which might indicate their functional association. Three further C/D box sRNA genes overlap with genes for putative rRNA methyltransferases (sRNA5, sRNA11, sRNA17) and one gene (sRNA25) overlaps with a putative rRNA pseudouridine synthase.

The C/D box sRNAs26 contains an inverted order of the conserved boxes with the box D upstream of box C. This observation could be an effect of circular box C/D evolution that has been recognized in, for example, *Pyrococcus furiosus *[28].

**Figure 6:**
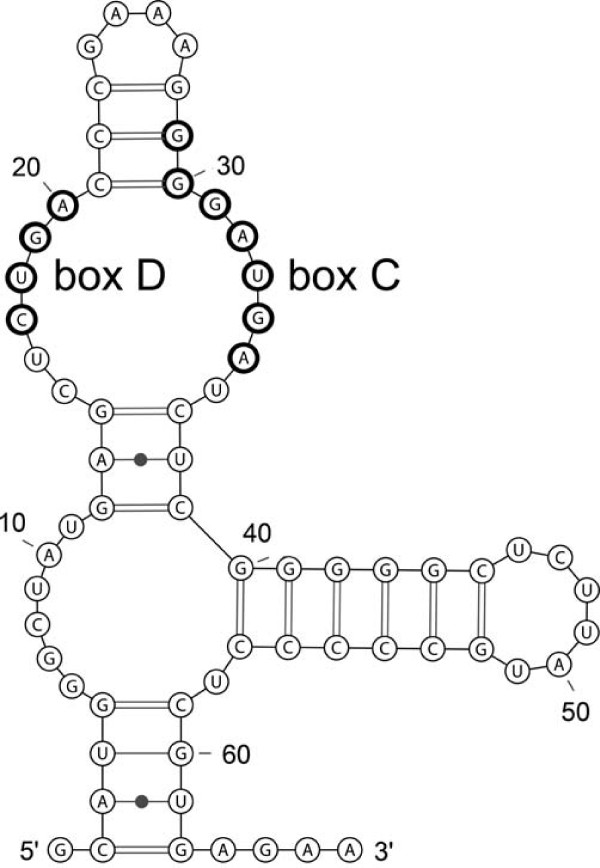
**Structure prediction for the tRNA^Met ^intron**. The predicted secondary structure of the stable and abundant tRNA^Met ^intron indicates the formation of a box C/D sRNA. The consensus box D and a box C motif with a T to G mutation at position 30 are indicated.

A single 159 nucleotide long H/ACA box sRNA was identified in the intergenic region between the genes NEQ389 (tyrosyl-tRNA synthetase) and NEQ392 (hypothetical protein). A H/ACA box sRNA guides the pseudouridylation of rRNA targets. The identified compact H/ACA box sRNA contains two extended hairpins that each contain a bulge with kink-turn (k-turn) bulges (Figure 5). Similar bulges were found in pseudouridine guide RNAs in *Pyrobaculum *[29]. A proper H domain (ANANNA) is not observed as the two hairpins are separated only by the sequence ACA in the hinge region (Figure 5). Potential pseudouridylation targets within the 16S rRNA and 23S rRNA were determined by the RNAsnoop algorithm [30] (Additional file 2). A stable RNA was identified to be encoded in the 66 nucleotide intron of the elongator tRNA^Met^. This tRNA is one of four intron-containing tRNAs but the other three introns are considerably shorter. The RNA contains elements that suggest its potential role as a C/D box sRNA potentially guiding RNA methylation (Figure 6). The introns of tRNAs are usually rather unstable products and previously only tRNA^Trp ^species were known to contain a functional C/D box sRNA in few archaea [31,32]. The *N. equitans *tRNA^Met ^intron also contains a postulated stable hairpin with six consecutive GC base pairings. As split tRNA genes might have been a result of the split of intron containing tRNAs, such a structure could hint at the origin of the GC-rich complementary sequences at the borders of *trans*-spliced tRNA halves.

### Mobile C/D box sRNAs

Alignment of the DNA stretches upstream and downstream of the identified small RNA termini enables the analysis of potential promoter and terminator elements. Previously, the conserved elements of nanoarchael tRNA and tRNA half gene promoters were identified [9]. The promoters contain a clearly identifiable box A motif (5'-TTTAAA-3') 26 nucleotides upstream of the transcription start and the terminator contains a stretch of polypyrimidines (T-stretch) downstream of the tRNA gene. Both elements are described to be commonly employed by the archaeal RNA transcription machinery [22,33] and can also be found for the H/ACA box sRNA. Transcription and processing of C/D box sRNAs is more diverse. Some C/D box sRNAs (sRNA12, 13, 15, 16, 21) contain their own promoter and termination signals and transcription starts with a purine residue. However, most C/D box sRNAs do not contain easily identifiable promoters or start with a pyrimidine residue, which indicates that they are processed during maturation. Interestingly, potential dicistronic tRNA-sRNA precursors were identified. The gene for the most abundant C/D box sRNA, sRNA8, lies immediately downstream of the gene for tRNA^Val^. Therefore, 3' processing of the sRNA8-tRNA^Val ^precursor by RNase Z (NEQ064) automatically generates the 5' terminus of C/D box sRNA (Figure 5). This is, to the best of my knowledge, the first time that this processing activity has been observed in prokaryotes as tRNA-snoRNAs were previously thought to be unique to plants [34]. Furthermore, two tRNA^Gly ^isoacceptors (tRNAGly(CCC), tRNAGly(TCC)) are located adjacent to the C/D box sRNAs3 and sRNA14.

**Figure 7:**
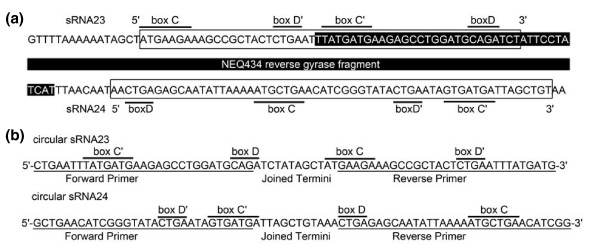
**C/D box sRNAs flank a split reverse gyrase gene**. **(a) **The gene for the amino-terminal portion of the split reverse gyrase (NEQ434, black) is flanked by two box C/D sRNAs. Box C and box D elements (boxed) are indicated to highlight the permutation of the sRNA24. **(b) **Sequencing of inverse RT-PCR amplificates with the indicated (underlined) outward facing primers revealed circular C/D sRNAs23 and 24. The detected sites of circularization indicate that circular C/D sRNAs are slightly larger than the average length of the linear molecules amplified by RNA-Seq methodology.

Most C/D box sRNAs overlap with mRNA sequences (Additional file 2). One notable feature is the occurrence of C/D box sRNA genes at the borders of split genes. They are found adjacent to NEQ156 (RNA polymerase subunit B, carboxy-terminal part), NEQ096/NEQ097 (hypothetical protein, carboxy-terminal part), NEQ124 (archaeosine tRNA-guanine transglycosylase, amino-terminal part) and NEQ434 (reverse gyrase, amino-terminal part) (Figure 7a). NEQ157, the gene located at the position where the amino-terminal portion of RNA polymerase subunit B would be expected for a continuous gene, is flanked by two C/D box sRNA genes. In addition, the amino-terminal portion of the reverse gyrase is also flanked by two C/D box sRNAs. In eukaryotes, snoRNAs are recognized as mobile genetic elements [35] that often associate with introns. In archaea and potentially in *N. equitans*, C/D box sRNA can be located within tRNA introns [36]. Future research will need to determine the basis for C/D box sRNA mobility in Archaea. The observation that one of the two C/D box sRNAs that flank the reverse gyrase half gene is permuted (Figure 7a) hints at a circularized intermediate structure. An inverse RT-PCR approach was employed to search for circular C/D box sRNAs. Both the C/D box sRNA23 and the permuted C/D box sRNA24 were detected as circular molecules in the cell (Figure 7b). Only the circular arrangement of the permuted C/D box sRNA24 allows the correct, conserved order of the C and D box elements and might therefore guarantee functionality. This phenomenon needs to be considered for automated genome annotation procedures of C/D box sRNAs. Genome rearrangements that create permuted C/D box sRNAs might only be detectable for recent fragmentation events as inactive C/D box sRNA genes will quickly accumulate mutations. Taken together, these observations strongly suggest an involvement of C/D box sRNA sequences in the splitting of both tRNA and protein encoding genes in the fragmented genome of *N. equitans*.

## Conclusions

In the presented study over 12 million RNA sequence reads were mapped to the minimal 0.49 million bp genome of *N. equitans*. The resulting sequencing depth allowed the detection of all predicted tRNA half precursors of the tRNA *trans*-splicing pathway. In addition, further evidence for the currently unique absence of RNase P in this organism was obtained. The analysis of the abundant small RNA population identified a considerable fraction of crRNAs as well as C/D box and H/ACA box sRNAs. These findings underline the importance of these two RNA fractions for an organism that lost most essential pathways for the synthesis of amino acids, nucleotides, cofactors, and lipids. It seems plausible that an organism that relies on the import of nucleotides would require their usage to be constrained. Nevertheless, crRNAs are abundant in the cell, which is in contrast to the silenced expression of CRISPR clusters found, for example, in some bacteria [37,38]. *N. equitans *appears to require the constant expression of this interference system against the attack of mobile genetic elements even though viruses of *N. equitans *are yet to be discovered. The abundant C/D box and H/ACA box sRNA fraction showcases the importance of rRNA processing events for *N. equitans *that is mirrored in its large set of RNA processing enzymes. These ribonucleoprotein complexes are suggested to ensure proper modification (and processing) of rRNAs in hyperthermophilic growth conditions. In conclusion, analysis of the RNA fractions in the minimal *N. equitans *cell revealed the loss or degeneration of universal RNA molecules (tRNAs, RNase P) while other, seemingly less essential RNA species (crRNAs, C/D box and H/ACA box sRNAs) are found to be highly abundant. The identification of C/D box sRNAs adjacent to split protein encoding genes and tRNAs as well as within a tRNA intron suggests their involvement in genome fragmentation.

## Materials and methods

### Cell cultivation and RNA isolation

*N. equitans Kin4-M *cells were a kind gift of D Söll. The organism was grown in the Archaeenzentrum Regensburg (H Huber, M Thomm, K Stetter) in a 300 liter fermenter in simultaneous culture with *I. hospitalis KIN4-I *and purified by gradient centrifugation as described [1]. Total RNA was isolated by SDS-lysis of the cell pellet and phenol/chloroform extraction as described [5] and small RNAs were purified from total RNA using the MirVana RNA extraction kit (Ambion (Paisley, UK).

### RNA-Sequencing

*N. equitants*/*I. hospitalis *RNA (3 μg) was treated with T4 polynucleotidekinase to ensure proper termini for ligation. A protocol for the dephosphorylation of 2'3' cyclic phosphate termini was modified from [39]: 1 μg of RNA was incubated at 37°C for 6 hours with 10 units T4 polynucleotidekinase and 10 μl 5 × T4 polynucleotidekinase buffer (NEB, Ipswich, MA, USA) in a total volume of 50 μl. Subsequently, 1 mM ATP was added and the reaction mixture was incubated for 1 hour at 37°C to generate monophosphorylated 5' termini. RNA libraries were prepared with an Illumina TruSeq RNA Sample Prep Kit and sequencing on an Illumina HiSeq2000 sequencer was performed at the Max-Planck Genomecentre, Cologne (Max Planck Institute for Plant Breeding Research, Köln, Germany).

### Identification of small RNA species

Sequencing reads were trimmed by (i) removal of Illumina TruSeq linkers and poly-A tails, and (ii) removal of sequences using a quality score limit of 0.05. A total of 16,614,433 reads with an average length of 62.3 nucleotides were obtained after trimming. Of these, 626,555 reads below 15 nucleotides were removed, and 12,178,737 reads were mapped to the *N. equitans *reference genome (GenBank: NC_005213) with CLC Genomics Workbench 5.0 (CLC Bio, Aarhus, Denmark). The following mapping parameters were employed: mismatch cost, 2; insertion cost, 3; deletion cost, 3; length fraction, 0.5; similarity, 0.8). This program was also utilized to determine the coverage of individual RNA molecules. All predicted RNA molecules and their termini were manually verified and all intergenic regions were checked for the presence of RNA molecules with coverage of less than 1,000 reads. The following algorithms were used for the computational analysis of the data: RNA folding (Mfold [40]), tRNA gene prediction (tRNAScan-SE [41]), snoRNA gene prediction (snoscan [25]), C/D box sRNA target prediction (plexy [26]), H/ACA box sRNA target prediction (RNAsnoop [30]), crRNA identification (crisprdb [18]), RNA alignments (ClustalW2 [42]), RNA visualization (VARNA [43]). Gene annotations were obtained from GenBank and tRNA annotations were taken from [17].

### Inverse RT-PCR

Circular C/D box sRNAs 23 and 24 were amplified from the small RNA purification sample via the Thermoscript RT-PCR system (Invitrogen (Paisley, UK) with Thermoscript reverse transcriptase and Platinum Taq DNA polymerase according to the manufacturer's instructions. The RNA was denatured at 100°C for 5 minutes and snap-cooled on ice for 5 minutes to facilitate reverse transcription at 70°C through potential secondary structures of the RNA. The following oligonucleotides were employed: sRNA23For, 5'-CTGAATTTATGATGAAGAGCCTGGATGCAG-3'; sRNA23Rev: 5'- CATCATAAATTCAGAGTAGCGGCTTTCTTC-3'; sRNA24For, 5'- GCTGAACATCGGGTATACTGAATAGTGATG-3'; sRNA24Rev, 5'- CCGATGTTCAGCATTTTTAATATTGCTCTCAG-3'. The oligonucleotides partly overlap to ensure proper annealing to the sRNA template. PCR amplificates were cloned into a pCR2.1 TOPO vector (Invitrogen) and subjected to DNA sequencing (Eurofins MWG Operon (Ebersberg, Germany).

### Data availability

The RNA-Seq data are available at NCBI's Gene Expression Omnibus (GEO) website as series GSE38821.

## Abbreviations

Cas: CRISPR associated protein; CRISPR: clustered regularly interspaced short palindromic repeats; crRNA: CRISPR RNA; PCR: polymerase chain reaction; RT: reverse transcriptase; snoRNA: small nucleolar RNA; sRNA: small RNA.

## Competing interests

The author declares that they have no competing interests.

## Authors' contributions

LR planned and initiated the project, analyzed the data, wrote the manuscript and approved the final version of the manuscript for publication.

## Supplementary Material

Additional file 1 ***N. equitans *mapped RNA reads**. Illumina HiSeq2000 sequencing reads were mapped to the *N. equitans *reference genome (GenBank: NC_005213, 490885 bp). The excel file contains the read coverage for the entire genome mapping. The genome region 449944 to 449989 (AAAAAAAGAAGAAAGAAAAAAGAAAGAAATAAAAAA) causes poly-A mapping artifacts. Click here for file

Additional file 2 ***N. equitans *C/D box sRNAs and H/ACA sRNAs**. This excel file contains detailed analysis of the C/D box sRNAs and H/ACA sRNAs. Indicated are genomic location, genomic context, termini, box C and box D structures and potential sites of action. Click here for file
